# Investigating the Impacts of a Modified Mindfulness Practice on Minoritized College Students’ Chronic Stress

**DOI:** 10.1089/imr.2024.0009

**Published:** 2024-07-31

**Authors:** Midley Michaud, Maiya Evans, Rebecca Mendez, Jalena Zapanta, Anthony Trochez, Kala M. Mehta, Leticia Márquez-Magaña, Audrey Parangan-Smith

**Affiliations:** ^1^ Department of Biology, San Francisco State University, San Francisco, CA, USA.; ^2^ SF BUILD, San Francisco State University, San Francisco, CA, USA.; ^3^ Department of Holistic Health, College of Health and Social Sciences, San Francisco State University, San Francisco, CA, USA.; ^4^ Department of Education, University of California Los Angeles, Occidental College, Los Angeles, CA, USA.; ^5^ Department of Epidemiology and Biostatistics, University of California, San Francisco, CA, USA.

**Keywords:** mindfulness practices, undergraduate students, race, ethnicity, perceived stress, STEM

## Abstract

**Context:**

Students of color in the United States experience elevated stress across the entire spectrum of education, spanning from early stages of K-12 to the more advanced stages of postgraduate studies. This sustained state of chronic stress decreases learning and curtails opportunities, especially in science, technology, engineering, and math (ST EM) fields, where stress levels are considered exceptionally high. Mindfulness-based practices such as MBSR have a proven effective for stress reduction in college students. However, to date, mindfulness practices have yet to be designed to support the unique needs of minoritized students with intersectional identities (e.g., poor, English as second language learners, and sexual/gender minorities) that are stigmatized in ST EM.

**Objectives:**

This article describes the development of an online, eight-week modified mindfulness practice (MMP) for minoritized students adapted from traditional MBSR. The MMP was purposely designed to be culturally inclusive and anti-racist, with the goal to reduce stress in undergraduate students of color in ST EM.

**Methods:**

In this pilot study, we assessed the impact of MMP using both biological and perceived stress measures. Specifically, cortisol was measured from donated biospecimen hair samples, the Perceived Stress Scale measured perceived stress, and key informant interviews were conducted to understand student stressors and coping strategies before and after the intervention.

**Results:**

While the observed decrease biological and perceived stress before and after the intervention was not statistically significant due to the small sample size of this pilot study, we see a dramatic positive change in student coping strategies.

**Conclusion:**

This study highlights the importance of providing minoritized students with options for stress reduction that are relevant and accessible.

## Introduction

Students of color in the United States experience elevated stress across the entire spectrum of education, spanning from early stages of grades K-12 to the more advanced stages of postgraduate studies.^1–3^ This sustained state of chronic stress decreases learning and curtails opportunities, especially in science, technology, engineering, and math (STEM) fields, where stress levels are considered exceptionally high.^4^ Furthermore, minoritized groups in STEM, including racial and ethnic minorities, women, and individuals with differential abilities, have been found to underperform in academic environments owing to factors such as worries about confirming negative stereotypes.^5,6^ Individuals with intersecting identities within these groups may receive a double-dose of stressors in these stigmatized domains.^7–10^

Undergraduate students in STEM disciplines report high rates of perceived stress.^4^ This is associated with several negative educational outcomes, such as decreased attendance, lower grades, matriculation, and applications to biomedical graduate study.^4^ In addition, people of color face complex stressors related to race and discrimination, immigration status, economic inequities, and other multifaceted societal factors.^11^ The COVID-19 pandemic exacerbated these stressors, especially among students of color. These hurdles included limited access to essential resources (e.g., personal computers and internet access) and the experience of residing in multigenerational homes).^11^

Mindfulness meditation is a centuries-old practice of being present and deliberately aware of one’s own breath, inner thoughts, feelings, and surroundings.^12^ It originates from Buddhism but can be taught in a secular manner. Mindfulness meditation has been shown to reduce stress, improve sleep, and cognitive functioning.^13^ Mindfulness-Based Stress Reduction (MBSR) is an adaptation of the practice of mindfulness meditation to health contexts to alleviate stress, foster awareness and relaxation, and improve quality of life.^12^ Mindfulness-based practices, including MBSR, have proven effective for stress reduction in college students.^13^ However, to date, mindfulness practices have yet to be designed to support the unique needs of minoritized students with intersectional identities (e.g., poor, English as second language learners, and sexual/gender minorities) who are often stigmatized in STEM.^14^

We describe the development of a modified mindfulness practice (MMP) adapted from an 8-week validated MBSR intervention for minoritized students. The MMP was purposely designed as a pilot study to be culturally inclusive, anti-racist, and to test its ability to reduce stress among undergraduate students of color in STEM. Although previous studies show that all undergraduates in STEM encounter higher levels of stress than in other fields,^4^ we predicted that minoritized students in STEM encounter additional levels of stress owing to historic structures of racism in education. Therefore, this study is one of the first interventions tested to combat both academic stress in STEM and racism stress in higher education.

## Materials and Methods

### Context

This pilot study is part of the SF BUILD (Building Infrastructure Leading to Diversity) project funded by the National Institutes of Health BUILD award.^15^ SF BUILD is a partnership between San Francisco State University and the University of California San Francisco (UCSF) to implement and study interventions to enhance diversity of the biomedical research workforce. To date, 102 undergraduate scholars have participated in the SF BUILD training program over 9 years. Scholars are often students of color, from historically minoritized communities, Pell grant eligible and from families below the median socioeconomic status. The MMP study employs a mixed-method approach, including the collection of biospecimens to assess biological stress, measurements of perceived stress, and key-informant interviews for in-depth insights.

### Recruitment

Student participants were recruited during a weekly online professional development class. A recruiter addressed questions about the hair collection process, analysis and sample access, clarified misconceptions, and addressed knowledge gaps regarding the use of biospecimens.^16^ A pamphlet and video on hair-collection with people of color was created and shared. To reduce participant stress and anxiety around biospecimen collection, study participants choose someone they trusted to cut their hair. Approval for human subject research was obtained from the Institutional Review Boards at the California State Committee for the Protection of Human Subjects and UCSF.

### Modified Mindfulness Practice

The mindfulness intervention was a one-arm pilot intervention with no control condition conducted online in the Spring 2022 semester. The MMP we developed and implemented was rooted in MBSR. The full MBSR, developed by John Kabat-Zinn, has been described previously.^17^ MBSR-based mindfulness practices were chosen because they are not affiliated with any particular religion, training manuals to implement this practice are available, and there is extensive literature demonstrating its efficacy in high-stress populations. The MMP instructor was formally trained in an MBSR-based practice and is certified in holistic health studies.

For this pilot study, MBSR mindfulness practices were modified to be culturally relevant and accommodate the needs of participants who were undergraduate students of color in the SF BUILD training program. The traditional MBSR day-long silent retreat was not incorporated owing to constraints in the undergraduate schedule. Additional modifications to the MBSR mindfulness practices included integrated cultural social support relevant to the participants. In traditional MBSR, culture is often left out of the conversation, which likely limits its effectiveness with some minoritized groups. For this intervention, it was important that cultural relevance was considered as a valuable component of the mindfulness training.

### Adapted curriculum

The MBSR curriculum used in this study was adapted from the Authorized Curriculum Guide^17,18^ using approaches described by Cheryl Woods-Giscombé.^14^ Specifically, a woman of color leads the MMP sessions to improve instructor immediacy and build trust.^19^ Students participated in 1-h MMP sessions once a week for 8 weeks. Different areas of MBSR were covered each week (i.e., body scan meditation, sitting yoga, walking meditation, deep breathing) and the curriculum was modified based on student feedback. For example, the practice was shortened because students reported that some of the meditations were too long. Similar to the Woods-Giscombe study,^14^ weekly lessons were adapted to be more culturally relevant for the students. Students were shown videos featuring people of color and mindfulness practices with techniques borrowed from indigenous and tribal traditions. Students were invited and encouraged to share experiences from their personal cultural experiences, and to discuss feelings or ideas pertaining to race, ethnicity, nationality, and/or culture. Students were encouraged to continue mindfulness practices learned that week at home.

## Measures

### Biospecimens

Biospecimens collection occurred in March and May 2022. For this pilot study, hair collection kits were mailed to the student participants. The kit included instructions for self-collection of hair, an index card, scissors, scotch tape, rubber bands, sanitizing wipes, and a postage-paid, self-addressed return envelope for biospecimens. Participants were instructed to cut a pencil-width section of hair from the posterior side of the skull and use scotch tape to attach the hair sample to the index card. Each index card had a unique identifier to link the hair biospecimen to the participant. The literature suggests that hair maintains a good record of cortisol levels over time; 10 mg of hair sampled approximately 2–3 cm from the scalp should represent 2–3 months of growth and cortisol exposure.^20^ Hair samples were stored in sealed envelopes at room temperature until processed by researchers. We followed previously described standard methods for hair processing and cortisol analysis.^21–26^

### Optimization of cortisol assay using hair samples

Cortisol measurement in hair requires optimization of the standardized protocol to achieve a low coefficient of variation (CV) in the results obtained. We used the Cortisol Enzyme-Linked Immunosorbent Assay (ELISA) kit manufactured by ALPCO which typically yields results with a low CV (up to 20% per manufacturer’s specifications). The CV for the cortisol levels found in this study ranged from 9% to 11%. The Intra-Assay CV is 9% and the Inter-Assay CV is 11%. These results suggest that the methods optimized in this study for the organic extraction of cortisol from hair, evaporation of the solvent, and resuspension of the extracted materials are suitable.

**FIG. 1.**
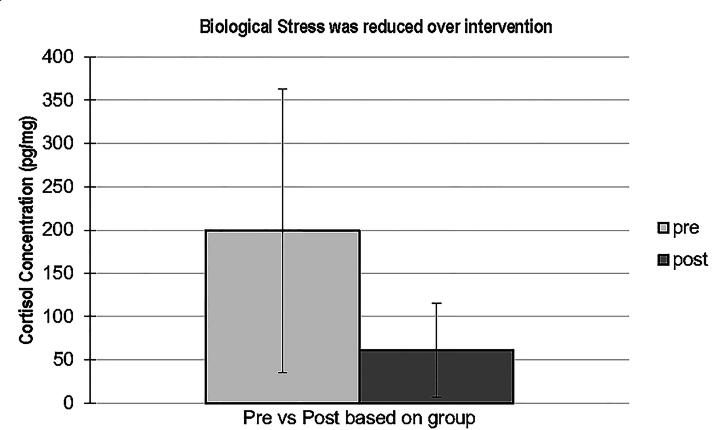
Mean cortisol concentrations pre- and post-MMP intervention. Cortisol levels in hair were collected from six MMP participants pre- and post-MMP intervention. Error bars represent standard deviation. MMP, modified mindfulness practice.

Using our optimized protocols for cortisol extraction, the amount of cortisol measured in the three hair samples analyzed in the study ranged from 20 to 379 pg/mg of hair (Fig. 1). This broad range has been found in several studies of cortisol levels in hair including average cortisol concentration ranges from 7.7 to 224.9 pg/mg in a control group of healthy participants and 10.8–295 pg/mg in individuals with various diseases.^27,28^ A recent study showed similar cortisol levels in a multiethnic sample.^29^

### Perceived Stress Scale

**FIG. 2.**
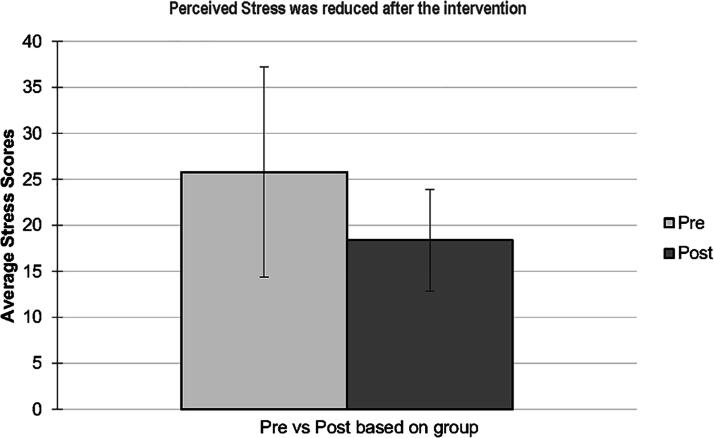
Average PSS scores pre- and post-MMP intervention. PSS scores were collected from six MMP participants at pre- and postintervention time points. Scores were calculated based on the 10-items PSS from the survey. The average of the scores was used to plot the graph. Error bars represent standard deviation. PSS, Perceived Stress Scale.

Self-reported stress data were collected using the Perceived Stress Scale (PSS). This 10-question scale assesses incidents of stressors over the past month and were analyzed (Fig. 2) as described.^30^

### Qualitative probes

Key interviews were conducted to understand the effects of the MMP intervention on coping strategies and the overall well-being of undergraduate students. Participants completed the structured, 30-min interviews before (week 0) and after (week 8) the MMP intervention. These interviews consisted of five questions regarding experiences of stress and coping strategies. All interviews were recorded and transcribed via Zoom.

### Data analysis

#### Analysis of biospecimens: cortisol levels

Cortisol levels in hair were measured for long-term stress exposure (1–3 months of hair growth) using an immunoassay (ELISA) initially developed for cortisol measurements in saliva (ALPCO, Inc). Hair sampling and analysis were performed in triplicates from a single extraction. We used a five-parameter linear regression analysis (Gen 5 version 2.04) to determine the cortisol concentration in nanograms per milliliter before correction for hair weight. The detection limit of the ELISA assay was ∼1 ng/mL, with a 95% confidence limit. The resulting hair cortisol concentration for each sample was converted into pg/mg using the following conversion formula:

$$\frac{\left[\left(\text{ng}/\text{mL}\right)\;\times \;\left(0.150\;\text{mL}\right)\;\times \;\left(1\;\text{mL}/0.8\text{mL}\right)\right]}{\left(100\;\text{mg}\;\times \;1000\right)}$$

This equation included the weight (10 mg) and volume of the reconstituted hair sample (0.150 mL) and a correction factor that accounted for the initial methanol volume (1 mL) before the extraction step and the portion used (0.8 mL) during the evaporation step (Dr. Xiaoling Song, by communication). Changes in biological stress (or cortisol concentrations) were evaluated using paired *t* tests.

#### Analysis of the PSS

The impact of MMP on perceived stress was measured by analyzing the difference between participant pre- and post-PSS scores collected at weeks 0 and 8. The PSS scores were obtained by reversing responses to the four positively stated items and adding the scores for each item. The average change in PSS was evaluated using paired *t* tests given that results from the Shapiro–Wilks test suggest normal distribution.

#### Analysis of key informant interviews

The first round of coding for interview themes was conducted by a single researcher (M.M.). This led to the development of a codebook used in discussions by the research team (M.M., A.T., R.M., M.E., K.M., A.P.S.) to attain consensus with each round of coding. Data were subjected to inductive thematic analysis, which permitted assessing the data, identifying patterns, concepts, and ideas. To facilitate the production of high-quality findings, a categorization process^31^ was used to organize data into groups of interconnected codes. This theme development method is described in Vaismoradi and Snelgrove.^32^ A conceptual model was created to explain relationships among main themes using a Grounded Theory approach.^33^ Codes were quantified to determine percent coverage within themes and representativeness across interviews. Theme saturation was identified and coded through a comparative analysis. Although interpretation was not verified with interviewees, credibility was obtained by applying theory-grounded interpretation, an accepted qualitative method.

## Results

### Sample characteristics

Six students consented to the MMP pilot study, and three participants donated complete pre- and posthair samples (Table 1). The mean participant age was 23 years (standard deviation [SD] = ± 8.9), and the majority (66%) were men. All (100%) participants interviewed identified as BIPOC, including self-identification as Latinx, Pilipinx, Vietnamese, and Burmese, were seniors and completing one of the following majors: Psychology, Biochemistry, Microbiology, and Computer Science.

**Table 1.** Demographic characteristics of the study participants.

Characteristics	Mean (SD) or *N* (%)
Age (mean +/− SD)	23 +/− 8.9
Sex	
Female	2 (34%)
Male	4 (66%)
BIPOC^a^	6 (100%)
Class Standing Senior	6 (100%)

^a^ Black, Indigenous, Person of Color.

### Biospecimen results

A reduction in biological stress was observed as measured by the mean cortisol concentration before and after the MMP intervention (Fig. 1). The preintervention cortisol concentration exhibited a mean of 199 pg/mg, SD ± 164, and postintervention cortisol concentration displayed a mean of 61 pg/mg, SD ± 54 (*p* = 0.2404).

## PSS Results

Similar to changes in biological stress levels before and after the MMP intervention, a reduction in perceived stress measured by the PSS survey was also observed (Fig. 2). Average PSS scores were M = 26, SD ± 11 at preintervention and M = 18, SD ± 6 at postintervention (*p* = 0.2297).

### Themes from key informant interviews

Four critical themes emerged from participants’ interviews: A. Common stressors B. Reported stress; C. Community cultural support; D. Coping strategy. These key findings were identified as the main factors influencing how participants experience and cope with stress. Table 2A–D highlights student quotes aligned with each theme. Examples from themes A–C did not differ before and after the intervention.

**Table 2.** Themes That Emerged from Participants’ Interviews Regarding Stress, Pre-, and Post-MMP Intervention

A. Common stressors		B. Reported stress	
Pre-intervention	Post-intervention	Pre-intervention	Post-intervention
“My dad’s neighborhood has been **gentrified**. Our neighbors, they are all white now. Although it is not like in-your-face **racism**, it is **subtle cues**. If I go into the house and the neighbors are outside, they will give you some looks, like, what are you doing here?”	“You know how you might feel some **racial stress** coming from other people, but I think some are internalized. Because of working in more of a white people lab-based mostly with white people, I think I stress myself out about that, and kind of get what is called **imposter syndrome**.”	“The **anxiety**, I could fall over like I feel like my **chest gets tight** like it’s, not that I have a hard time breathing, but I can feel it in my **chest feels so tight**. I **can’t sleep** when I’m anxious. It feels sick even eating, **lost appetite**... I felt **lightheaded**.”	“How I experience it it’s like a lot of like the first thing I definitely think of is like when I experienced like lots of **anxiety**, lots of **no sleep**.”
“I’ve dealt with **racism** because I wasn’t born here. I was born in Burma... So, moving to the US... trying to find a home is hard, trying to find where you belong is hard because of the **language barrier** and your accent.”	“People **discriminate**. it’s the same. Never change. It’s just **racism** in general, I feel like towards Latinos mostly like **discrimination** like not letting you in here or there just because you are Latino.”	“When it gets too **stressful**, I mean I kind of feel like a **pain in my chest**.”	“Stress for me personally in my body feels like I’m very **tense**. I have to remember to relax my shoulders and unclench my jaw. I get **headaches**; that’s how I know I’m usually stressed I get stress headaches. I **can’t sleep** well or **have trouble sleeping** because I’m **stressed**.”
“The first real bit of **racism** I experienced was at SFSU… where I’ve been called the **N-word** before... this white person... he just came up to us and called us the **N-word** like hard R.’’	“Most of my stress comes from my **parent’s pressure**... I’m a **first-generation** college student. The **pressure to succeed** in life, to get a master’s, get a bachelor’s, or a Ph.D. That’s one of the biggest stresses in my life, trying to succeed or live up to my parent’s expectations. And school is very **stressful**.”	“When it gets too stressful, I feel like a **pain in my chest**… I would often, you know, **feel dizzy** and get **headaches**.”	“I get those **anxious**, scared feelings I’m just like I’m just **stressed** I’m just like I’m just **tense** about the situation.”

C. Community Cultural Support		D. Coping Strategy	
Pre-intervention	Post-intervention	Pre-intervention	Post-intervention
“We live in a very **Latino community**. We live in the Mission, so I feel like I don’t experience much racism or sexism here because everyone looks like me, and I know everyone. But it’s different when I visit my dad and his neighbors.”	“It’s very different compared to when I was growing up. growing up in Vallejo, you knew these things existed; when you grow up in a **diverse community**...it wasn’t like a problem to communicate with one another. No one was racist in that aspect.”	“It’s just mainly me trying to make time stepping away from all the problems and kind of doing what I enjoy like **listening to music** or **playing video games**.”	“I’ve been trying out the **body scan** assessment introduced in the SF build meetings we have every week. It works effectively in trying to **calm my senses down**.”
“I live in a predominantly you know **Asian neighborhood**, so I haven’t experienced a lot of racism. But from what I hear from my family in high school or especially my sister. She always experiences the average ignorant comments made by white people you know especially when they do not know about a certain culture.”	“I’ve learned how to look over that and I just like focus on myself, **focus on people who are close to me, focused on people who are supporting me**. And that definitely helped with like the whole racism stuff because like now I’m proud to be like what I am.”	“Going on **my phone playing games** or just **watching TV** just to ignore, like all the work I have.”	“There is a lot I learned in the sessions with Maiya. I like the **body scan**, and the **four-by-four breathing** is nice. I use that one the most probably once a week.”
“I made that clear that I don’t want a white man like I don’t want that, and the fact that they came up with like this person of color who is like **reminiscent of me** like helped me a lot.”	“A big factor that helps me a lot is that my **therapist** is **Filipino**. My mom’s Filipino, my dad’s Vietnamese, I grew up in a **traditional Filipino household**. I have like a therapist who **identifies with the same thing as me.** I don’t have to explain a lot. She gets me in a way I wouldn’t have to explain to a white person.”	“Making **jokes** and making people comfortable to talk to me, that’s how I dealt with stress.”	“**Four-by-four breathing** exercise. When I feel my chest is tight, I do a **breathing exercise** or **journaling** to stay grounded.”

The bold text reflects keywords from participant interview responses that resulted in the development of the four themes.

A. Common Stressors, B. Reported Stress, C. Community Cultural Support, and D. Coping Strategy.

1) Common stressors

The most common stressor reported besides school was racism in academia and personal life. Students are also burdened with financial and family responsibilities, including finding a job after college, being uncertain of the future, and parents’ pressure and expectations, which added to their stress.

2) Reported stress

When students were asked about their physiological responses to stress, the most common responses were trouble sleeping, anxiety, headaches, and chest pain.

3) Community cultural support

Students were asked about coping strategies to stress caused by experiences of racism. Relying on their support system and being surrounded by their community was most commonly reported.

4) Coping strategy

Before the MMP intervention, many students reported using coping strategies such as watching shows, playing video games, or YouTube videos as a form of distraction. After the MMP intervention, most students mentioned that their long-term healing from stress strategies consisted of mindful breathing exercises, body scans, mindful journaling, and mindful walking to stay grounded.

## Discussion

College students of all racial and ethnic groups experience high levels of stress, which can decrease learning and worsen health.^11^ Previous research has demonstrated the benefits and improvements in learning and health from mindfulness practices among various high-stress populations, including African Americans,^34,35^ surgical interns,^36–38^ and college students.^13^ However, it is important to highlight that mindfulness practices have yet to be studied in undergraduate students of color with intersectional identities who are stigmatized in STEM.

This pilot study showed the benefits of adapting MBSR-based practices for minoritized students in STEM. The MMP intervention had positive effects on student perceived and biological stress. The largest impact observed was a shift in student coping strategies. Postintervention, students highlighted that the use of breathing exercises, body scans, mindful journaling, and mindful walking was beneficial to them in coping with stress. Interestingly, students reported the importance of family support, community, and their cultural identity in coping with racism. This observation aligns with social and familial capital described in the Community Cultural Wealth model developed by Tara Yosso.^39^ BIPOC students bring in assets from their community to persist and navigate higher education.

It is important to note that this pilot study was conducted in 2022 during the COVID-19 pandemic which may have added potential stressors such as financial stress, limited social interaction, and challenges navigating online courses impacted students’ willingness to participate. Worries about the social and political landscape (i.e., racial and gender politics, increasing restrictions on reproductive rights, etc.) could have impacted our overall results. In fact, a recently published brief highlighted higher level of stress owing to racial injustice experienced by minoritized undergraduate student researchers during this time period.^40^ It is worth noting that participants continued to encounter and report stressors before and after the intervention, as indicated by the qualitative data. However, despite ongoing exposure to stressors, there was a noticeable reduction in both biological and perceived stress levels. This observation underscores the interplay between our quantitative findings and the qualitative data, indicating that stress reduction occurred despite the persistence of stressors reported by the participants.

Culturally responsive mindfulness practices have not been largely implemented in university settings. Our study is likely the first to show their utility in reducing the unique stress experienced by minoritized students in STEM. For example, we found that minoritized students could better engage with MMP principles when given the opportunity to discuss how racial and economic class structures impact access to mindfulness practices. When students were encouraged to see self-care practices as a universal human right rather than an extracurricular activity for the privileged class, students recognized how mindfulness is applicable to their lived experience. Future work using MMP in a college setting should include sharing a systematic inventory of wellness resources with participants, including resources that are part of campus services. Further studies on the impacts of MMP on diverse ethnic groups and other cultural backgrounds (e.g., Black/African Americans, Pacific Islanders, Latinx/Hispanics) need to be explored. Offering mindfulness interventions to other professional development programs could also help expand the mindfulness field in a positive and meaningful way.

Some limitations of the study deserve comment. This pilot study had a small sample size, thus impacting statistical significance and suggested inferences should be considered preliminary. As such, the positive effects observed on student perceived and biological stress were not statistically significant. This was not a randomized intervention. Rather, it was a convenience sample obtained through an undergraduate training program and there was no comparison group. Nonetheless, the descriptive and qualitative information gathered about MMP for minoritized students in STEM demonstrated the potential for positive effects on stress, including improved coping strategies, and contributes to ongoing work to improve the well-being of students who are understudied.

## Conclusion

This pilot study supports the idea that MMPs tailored to minoritized students in STEM can reduce their stress by adding to their coping strategies. Although cortisol results did not reach statistical significance in our small sample, students experienced a decrease in biological and perceived stress over the MMP period. Students reported that they adopted many of the mindfulness techniques they learned in the sessions for their own personal use. For example, students reported that they may now choose to use a breathing or meditation practice instead of watching television or social media to reduce stress. STEM disciplines have been shown to be stressful, especially to women and students of color. During the intervention, students of color disproportionately experienced additional stress due to COVID-19 and the impact of racism on the socio-political climate. Our results highlight the importance of providing minoritized students options for stress reduction that are relevant and accessible.

## Abbreviations Used

ELISA: Enzyme-Linked Immunosorbent Assay
PSS: Perceived Stress Scale
STEM: Science, Technology, Engineering, and Math (STEM)
